# Fish Feed Quality Is a Key Factor in Impacting Aquaculture Water Environment: Evidence from Incubator Experiments

**DOI:** 10.1038/s41598-019-57063-w

**Published:** 2020-01-13

**Authors:** Wenwen Kong, Suiliang Huang, Zhenjiang Yang, Feifei Shi, Yibei Feng, Zobia Khatoon

**Affiliations:** Key Laboratory of Pollution Processes and Environmental Criteria of the Ministry of Education, Key Laboratory of Urban Ecological Environment Rehabilitation and Pollution Control of Tianjin, Numerical Simulation Group for Water Environment, College of Environmental Science and Engineering, Nankai University, Tianjin, 300350 China

**Keywords:** Freshwater ecology, Environmental impact

## Abstract

The effect of fish feed quality has gained increasing attention to alleviate the harmful environmental impacts induced by intensive aquaculture. In current research, we have conducted an incubator experiment to highlight the effect of fish feed quality on aquaculture water environment. Fish feed from three manufactures with two different dosages (0.1000 g, 0.2000 g) was added to the culture medium with and without *Microcystis aeruginosa*. Treatments with *Microcystis aeruginosa* were named as MHT, MHP and MZT; while the treatments without *Microcystis aeruginosa* named as HT, HP and ZT. *Microcystis aeruginosa* densities and nutrients concentrations were measured in the study. Results have shown that fish feed quality (manufactures) has a great effect on nutrients concentrations in the absence of *Microcystis aeruginosa* (*P* < 0.05). Meanwhile, fish feed can stimulate *Microcystis aeruginosa* growth that is also influenced by fish feed quality excluding lag phase (0~12 day) significantly in general (*P* < 0.05). The maximum *Microcystis aeruginosa* density (*N*_*max*_) is 1221.5, 984.5, 581.0, 2265.9, 2056.8 and 1766.6 1 × 10^4^ cells mL^−1^ for MHT 0.1 g, MHP 0.1 g, MZT 0.1 g, MHT 0.2 g, MHP 0.2 g and MZT 0.2 g, respectively. In treatments with algae, fish feed quality affect total phosphorus (TP) concentrations (except the difference between MHT and MHP) and total nitrogen (TN) concentrations significantly (*P* < 0.05). For most of consumed nutrients, the obvious differences among all treatments were observed excluding lag phase in general (*P* < 0.05), which suggest that the nutrient utilization is also dependent on fish feed quality. Keeping in mind the above facts it is concluded that fish feed quality is a key factor in impacting aquaculture water environment.

## Introduction

Aquaculture is one of the fastest growing food producing sectors around the world. Global production of aquaculture increased from 4.17 × 10^7^ tonnes in 2000 to 8.0 × 10^7^ tonnes in 2016, and the annual growing rate reached 5.2% during this period^1^. Freshwater aquaculture is probably the most important form of aquaculture for the time being, and fish is by far the dominating product in freshwater aquaculture^2,3^. In fact, aquaculture production heavily depends on the external aquafeeds or nutrients supply to the aquaculture system^4^. Aquafeeds production has been widely recognized as one of the fastest expanding agricultural industries in the world^5^, and the annual growth rate of aquafeeds production reached 17% in China^6^. In 2018, total output of global aquafeeds was 40.1 million tonnes, of which Asia-Pacific’s aquafeeds production reached 28.5 million tonnes^7^. In practice, fish feed is the most important kind of aquafeeds with China being the top 1 in the world production of the fish feed^8^.

Currently, the rapid development and low entry barriers for China’s feed industry have led to the emergence of aquafeeds enterprises with insufficient conditions^9^. Meanwhile, production of carp and other omnivorous species is intensifying in China, and commercial aquafeeds enterprises are also being developed to serve these industries^10–12^. In 2017, there have been 6469 feeds manufactures in China, and 3145 feeds manufactures’ output are lower than 1 million tonnes^13^. Due to different production levels of fish feed producers, the fish feed qualities significantly differ both imaginably and practically. In Soong *et al*.’s^14^ study, although all grouper fish feed meal produced by 30 manufacturers can be used to feed grouper fish, the nutritional indicators and quality of these feed meals are not the same.

Quality analysis of fish feed is mostly founded on the growth rate of fish^15,16^, quality benefit of fish^15,17,18^, feed coefficient^19^ and so on^14,20^. In order to healthily promote the development of fisheries, some standards in US, Europe and China for fish feed have been formulated, such as “Nutrient Requirements of Fish and Shrimp” published by American National Research Council^21^ and regulation EC NO.767/2009 issued by European Parliament and Council^22^. In China, the Ministry of Agriculture has issued 21 standards for aquafeeds industry, and AQSIQ (General Administration of Quality Supervision, Inspection and Quarantine of the People’s Republic of China) and the National Standards Management Committee have issued 7 national standards for aquafeeds^23^. In these standards, nutritional indicators are primarily protein, crude fat, crude ash, calcium, phosphorus, lysine and so on. Moreover, these indicators are mainly following the “lower limit rule” rather than the specific contents, and actually they are still incomplete for fish feed and detailed ingredients of fish feed. Thus, fish feed quality from different producers may be still different even if they all meet the standards.

Despite huge potential benefits of aquaculture development, there are always concerns about its environmental impacts^24^. Recently, concerns on both fish feed quality and effects of fish feed on the aquaculture water environment have been elevated to a new level^25–31^, and the environmental effect of cage farms is believed to be a critical part for sustainable aquaculture^32^. Wasted fish feed in Ballester-Moltó *et al*.’s assays were estimated in the range of between 8.52% and 52.20% in aquaculture water bodies^29,30^. Edwards also believed that when harvesting fish, only about1/3 of the nutrients in the feed were removed, and 2/3 of nutrients were voided by fish during the growth process^27^. In general, 57% of the total feed nitrogen (N) and 76% of the total feed phosphorus (P) for fish lost to the environment, respectively^33^. However, most discussions about the effects of different fish feed on nutrients (N and P) enrichment did not consider the difference of fish feed quality^27,32,34^. In fact, uneaten fish feed or fish excretion from aquaculture activities release major macronutrients like nitrogen and phosphorous^33^, the released nutrients concentrations from uneaten fish feed of different quality or manufactures are expected to be different.

Meanwhile, nitrogen and phosphorus released from aquafeeds are not only the basic ingredients incorporated in feed to achieve good growth of aquatic animals (e.g. fish, shrimp), but also required for algal growth in water bodies^28,32,35,36^. In fish ponds of feeding common carp (*Cyprinus carpio*), 57~71% N and 44~58% P came from the fish feed, and they can be accumulated in fish, plankton and benthic organisms^37^. Nutrients produced through aquaculture activities are rapidly assimilated by phytoplankton, and this results in low concentration of inorganic nutrients in the water column^38^. Li *et al*.’ study noted that the massive growing phytoplankton absorb DIN and DIP effectively^39^.

In addition, aquaculture activities not only stimulate algal growth but also affect phytoplankton communities^40,41^ along with deteriorating water quality and adversely influencing human health aquaculture activities^42^. Wu *et al*.’s results showed that the release of N and P from fish feed stimulated algae growth^43^. Algae densities increased with increasing fish feed dosages in moderate nutrients concentrations from fish feed^43^. In Huang *et al*.’s study, enclosures with fish feed have higher algae biomass than those without fish feed, and blue-green algae dominated phytoplankton communities in enclosures with fish feed^28^. With the rapid growth of marine aquaculture activities in the coastal areas of Weihai, China, cellular abundance of diatoms and dinoflagellates increased between 2006 and 2014^39^. Affected by organic enrichment and sediment resuspension by shrimp, a shift in species dominance from Diatoms and Dinoflagellates to green algae was observed in shrimp aquaculture ponds in Hugues *et al*.’s study^44^. In sum, although fish feed qualities were also not considered in Rahman *et al*.^37^, Wu *et al*.^43^ and Huang *et al*.’s^28^ studies, the effect of different fish feed quality on algae growth is worthy to be studied.

Polyculture of Chinese carp, using large amount of commercial compound freshwater fish feed, has been recognized as a traditional way of increasing nutrient utilization in freshwater bodies. Additionally, in many freshwater lakes in China, *Microcystis aeruginosa* (*M. aeruginosa*) is a common cyanobacterium of harmful algal blooms^45^. In light of the above facts, compound freshwater fish feed from three different manufactures were selected to investigate effects of uneaten fish feed with different qualities on aquaculture water environment, including the characteristics of nutrients release, the effects of different fish feed on *M. aeruginosa* growth and nutrients utilization by *M. aeruginosa* through incubator experiment.

## Materials and Methods

### Experimental materials

*M. aeruginosa* (cyanobacterium) was obtained from the Freshwater Algae Culture Collection of the Institution of Hydrobiology (FACHB-905), which belongs to Chinese Academy of Sciences. The algae were cultivated in an illumination.

Commercial adult fish feeds, named HT, HP and ZT, are selected according to their popularity in aquafeeds market, which are used for polyculture in freshwater bodies such as lakes, reservoirs, ponds and so on. In other words, they are more or less fish-friendly feeds. HT is produced by Huaian Tongwei Company Limited, and this company is a large-scale feed enterprise invested and built by Tongwei in 2001. Huaian Tongwei Company Limited mainly produces aquafeeds as well as animal feeds, and the aquafeeds are widely used in the mainland of China. HP is widely used in Hebei province, China, which is produced by Hebei Panda Feed Company Limited. The company is incorporated in 2013 which mainly produces aquafeeds along with animal feeds. ZT is produced by Zhongshan City Taishan Feed Company Limited incorporated in 2004 and is widely used in Guangdong province, China. In 2010, feed sales of Zhongshan City Taishan Feed Company were 170000 tonnes, of which 90000 tonnes were for aquafeeds. Retail price of HT, HP and ZT feed are 6.2, 7.5 and 7.7 yuan kg^−1^ when we bought online for experiment, respectively, and the price is free of transportation. We think the higher retail price of ZT fish feed is caused by transportation costs. These fish feeds were crushed and sieved through Taylor pore size of 0.85 mm before use. HT, HP and ZT fish feed contains TP with 13.41, 12.15 and 11.37 g kg^−1^ respectively, while contains TN with 49.70, 45.85 and 38.75 g kg^−1^ respectively by analysis^43,46^. Nutritional indicators of these fish feeds disclosed by their respective manufacturers are shown in Table 1. These indicators are different with the same usage of fish feed and we believe that the quality of fish feeds is also different.

**Table 1:** Nutritional indicators of fish feed.

Fish feed	Crude protein (%) ≥	Crude fiber (%) ≤	Crude fat (%) ≥	Crude ash (%) ≤	Calcium (%)	TP (%) ≥	NaCl (%)	Moisture (%) ≤	Lysine (%) ≥
HT	28.0	12.0	3.0	15.0	0.5–2.0	0.60	0.2–1.5	12.5	1.2
HP	28.0	12.0	3.0	18.0	0.5–2.0	0.80	0.^3−^1.5	13.0	1.3
ZT	20.0	17.0	2.0	15.0	≤2.0	0.50	≤2.0	12.5	0.9

HT, HT fish feed is produced by Huaian Tongwei Company Limited; HP, HP fish feed is produced by Hebei Panda Feed Company Limited; ZT, ZT fish feed is produced by Zhongshan City Taishan Feed Company Limited; TP, total phosphorus in fish feed.

### Algal pre-culture

*M. aeruginosa* were cultured in M-II culture medium for 15 days before the experiment. The M-II culture medium was prepared in deionized water with 100 mg L^−1^ NaNO_3_, 10 mg L^−1^ K_2_HPO_4_, 75 mg L^−1^ MgSO_4_ × 7H_2_O, 40 mg L^−1^ CaCl_2_ × 2H_2_O, 20 mg L^−1^ Na_2_CO_3_, 6 mg L^−1^ Fe·citrate × H_2_O and 1 mg L^−1^ Na_2_EDTA × 2H_2_O. The initial pH value was adjusted to approximately 8.0 with 0.5 mol L^−1^ NaOH and 0.5 mol L^−1^ HCl. The operational temperature and light intensity were 28 °C and 3000 lx for the experiment undertaken under light conditions. In comparison, the corresponding values during the period of darkness were 20 °C and 0 lx. The cycle of light and darkness comprised 12 h of light and 12 h of darkness.

The medium containing algae was collected and then centrifuged for 15 min at a speed of 3000 r min^−1^. After removal of the supernatant, the algae were rinsed with 15 mg L^−1^ NaHCO_3_ solution and then centrifuged again. After repeating the above procedure twice, the algae obtained via this procedure were cultured in M-II medium without nitrogen or phosphorus, the process was defined as starvation cultivation. Three days later, the algae would deplete the intracellular polyphosphate stores^43^.

### Experimental methods

Effects of different fish feed on nutrients release and algae growths were assessed using batch incubation experiments. In the experiment, 400 mL sterilized M-II culture medium without nitrogen and phosphorus was used, and weights of 0.1000 g and 0.2000 g of the three different fish feed (from different manufactures) were added into the media served as P and N sources with 1 L flask. Treatments without algae containing 0.1 g fish feed were named “HT 0.1 g”, “HP 0.1 g”, “ZT 0.1 g”, and containing 0.2 g fish feed named “HT 0.2 g”, “HP 0.2 g” and “ZT 0.2 g”, respectively. Meanwhile, treatments with algae containing 0.1 g fish feed were named “MHT 0.1 g”, “MHP 0.1 g”, “MZT 0.1 g”, and containing 0.2 g fish feed named “MHT 0.2 g”, “MHP 0.2 g” and “MZT 0.2 g” conforming to the treatments’ name, respectively. Duplicates were prepared. Flasks were shaken and their positions were changed at random three times a day. The initial algae density was 1.0 × 10^5^ cells mL^−1^.

During the experimental period (37 days), algal cell densities were counted every two days using a haemacytometer under a microscope^43,47^. Counting was performed three times per sample. Water sampling started 1 day after algae addition, and total phosphorus (TP), total dissolved phosphorus (TDP), total particulate phosphorus (TPP = TP-TDP), orthophosphate (PO_4_^3−^-P), total nitrogen (TN), total dissolved nitrogen (TDN), total particulate nitrogen (TPN = TN-TDN) and ammonia (NH_4_^+^-N) were also measured every two days. Concentrations of PO_4_^3−^-P, TDP and TP were determined via the persulphate digestion and ammonium molybdate spectrophotometric method^48^. NH_4_^+^-N was analyzed using the phenol-hypochlorite method^48^. TN and TDN were analyzed using the procedure of alkaline potassium persulfate digestion with ultra-violet light spectroscopy^49^.

### *M. aeruginosa* growth kinetics

Algal growth can be well described by (original) Logistic function^50–54^. However, this function does not satisfy the initial conditions of algal growth. A modified Logistic function was proposed by Huang *et al*.^49^ and it is as follows:

1 $$N=\frac{{N}_{max}}{1+{e}^{a-rt}}+{N}_{0}-\frac{{N}_{max}}{1+{e}^{a}}$$

where *N* (1 × 10^4^ cells mL^−1^) is the algae density at any time, *N*_*max*_ is the maximum algae density (1 × 10^4^ cells mL^−1^), *r*(d^−1^) is the intrinsic growth rate, *N*_0_ is the algae density at 0 day, and *N*_0_ is 10 × 10^4^ cells mL^−1^ in the present study, *t*(d) is time and *a* (−) is a constant. *N*_*max*_, *a* and *r* can be obtained by fitting Eq. (1) to experimental data.

Growth rates ${\mu ^{\prime} }_{c}$ (1 × 10^4^ cells (mL·d)^−1^) can be derived from modified Logistic function in Huang *et al*.’s^49^ study as follows:

2 $${\mu ^{\prime} }_{c}=\frac{d{\rm{N}}}{dt}=\frac{{N}_{max}r{e}^{a-rt}}{{(1+{e}^{a-rt})}^{2}}$$

The growth rate reached its maximal value ${\mu ^{\prime} }_{cmax}=\frac{r{N}_{max}}{4}$ (1 × 10^4^ cells (mL·d)^−1^) when *N* equals to half of *N*_*max*_^49,51,53,55^.

The formula of the specific growth rate from the modified Logistic function as shown in Eq. (3), describing variations of specific growth rates with time is also better than that derived from Logistic function^49^:

3 $${\mu }_{c}=\frac{d(lnN)}{dt}=\frac{{N}_{max}r{e}^{a-rt}(1+{e}^{a})}{(1+{e}^{a-rt})[({N}_{0}+{N}_{0}{e}^{a}-{N}_{max}){e}^{a-rt}+{N}_{0}+{N}_{0}{e}^{a}+{N}_{max}{e}^{a}]}$$

where ${\mu }_{c}$ (d^−1^) is defined as the computed specific growth rate.

### Statistical analysis

Experimental data was analyzed statistically by using Origin 8.6 and SPSS 19.0. Logistic model was examined for their fit to the experimental data using Origin 8.6. Origin 8.6 or SPSS 19.0 is used to determine correlation coefficients between the measured and predicted variables as well as between *M. aeruginosa* densities and nutrients concentrations. The statistical analysis is applied to identify the significant differences among groups with different fish feed by analysis of variance (ANOVA) with SPSS 19.0. Moreover, standard deviation was calculated and data was expressed in terms of means + SD of the two replicates.

## Results and Discussion

### Effects of different fish feed on nutrients concentrations without algae

#### Effects of different fish feed on phosphorus concentrations

**Figure 1:**
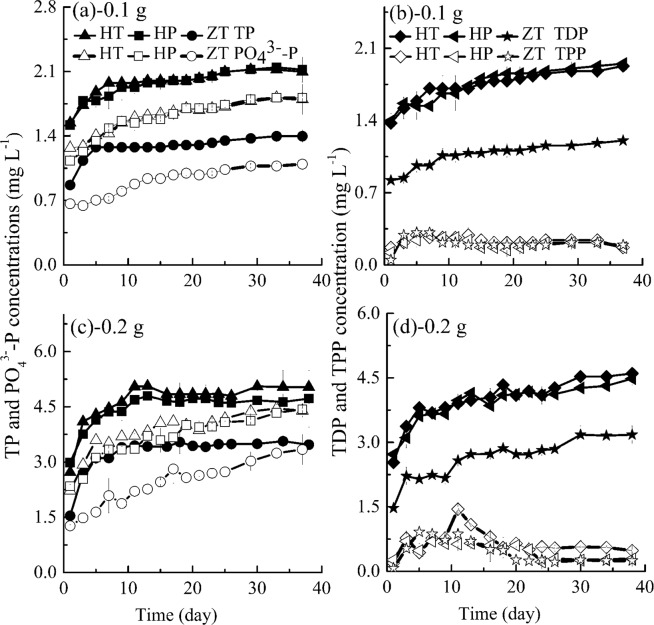
Variations of TP, TDP, TPP and PO_4_^3−^-P concentrations with time in groups without algae (HT, fish feed of HT; HP, fish feed of HP; ZT, fish feed of ZT). Data shown is the mean ± SD of two independent measurements.

Phosphorus is chemical compound found in fish feed^33^, its labile form (PO_4_^3−^-P) is a major form of released phosphorus from fish feed^43^. From Fig. 1, TP, PO_4_^3−^-P and TDP concentrations in treatments with HT, HP and ZT increase gradually in the first 10 days and then enter into a stable phase. Meanwhile, released concentrations of TDP and PO_4_^3−^-P from fish feed reached 85.39~90.00% and 75.23~89.91% of their corresponding maximal values at the first sampling day (or 24 hours). Akhan and Gedik’s research results also indicated that release of nutrients from fish feed occurred rapidly, they believed that uneaten fish feed should be removed quickly to avoid nutrient enrichment^32^.

Under same fish feed dosage, TP (TDP or PO_4_^3−^-P) concentrations in treatments with HT and HP feed are 1.33~1.66 times higher than those of ZT, which is not consistent with their nutritional indicator of TP (in Table 1). This may be because the TP indicator in these feeds just follows “the lower limit rule”. Calculated results shows that average TP concentrations are 1.97, 1.96 and 1.28 mg L^−1^, average TDP concentrations are 1.75, 1.74 and 1.06 mg L^−1^, average PO_4_^3−^-P concentrations are 1.60, 1.59 and 0.91 mg L^−1^ for HT 0.1 g, HP 0.1 g and ZT 0.1 g respectively, and these concentrations also doubles in treatments with 0.2 g correspondingly. This also implies that both HT and HP feed have much larger capacities in releasing phosphorus nutrients than ZT feed. In addition, significant analysis shows that there is a noteworthy difference in releasing phosphorus nutrients between HT and ZT and between HP and ZT (*P* < 0.001), while there is no significant difference between HT and HP (*P* > 0.05). Significant analysis also shows that fish feed dosage affects TP, TDP and PO_4_^3−^-P concentrations quite significantly (*P* < 0.001), which conforms to Wu *et al*.’s results^43^.

In Fig. 1(b,d), variations of TPP concentrations with time are quite different from those of TDP. In general, TPP concentrations in HT, HP and ZT groups are quite low and close to each other with the same dosage of fish feed, and all increase firstly and then decrease slightly. Fish feed quality does not have a significant effect on TPP concentrations in general (*P* > 0.05).

#### Effects of different fish feed on nitrogen concentrations

**Figure 2:**
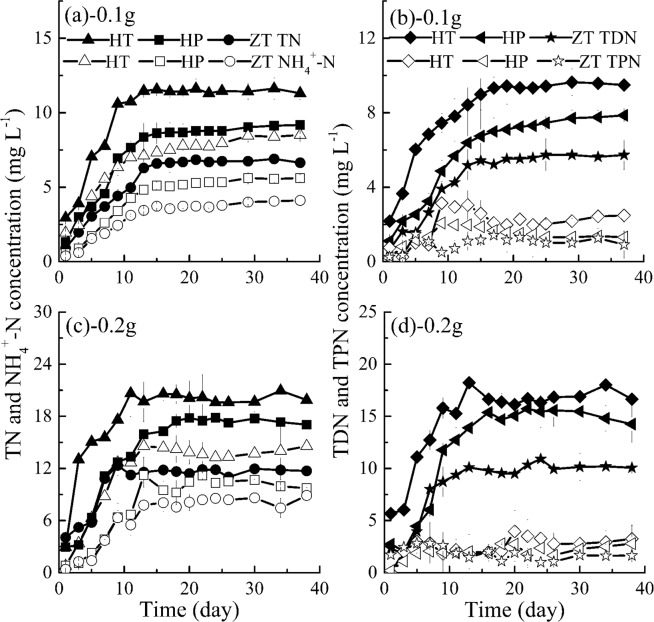
Variations of TN, TDN, TPN and NH_4_^+^-N concentrations with time in groups without algae (HT, fish feed of HT; HP, fish feed of HP; ZT, fish feed of ZT). Data shown is the mean ± SD of two independent measurements.

Uneaten fish feed is probably the major input of nitrogen to the aquatic environment^35,56–58^, and the nitrogen cycle in aquaculture ecosystem begins with the introduction of protein in fish feed and NH_4_^+^-N is a by-product of protein catabolism^26^. From Fig. 2, compared with the released process of phosphorus nutrients from HT, HP and ZT fish feed, nitrogen concentrations rise comparatively very slowly and the time to reach nitrogen nutrients equilibrium concentrations is much longer. TN, TDN and NH_4_^+^-N concentrations increase gradually in about 15 days, and then reach equilibrium in the following days. In addition, it is clearly observed from Figs. 1 and 2 that TN equilibrium concentrations are higher than TP equilibrium concentrations (1.40~5.04 mg L^−1^) in the present experiment. Fernandes *et al*. also observed that leaching loads of fish feed for the bluefin tuna were slightly high for nitrogen as 26 kg N tonne^−1^, but significantly low for phosphorus as 4 kg P tonne^−1^ ^25^.

As shown in Fig. 2, released TN, TDN and NH_4_^+^-N concentrations from different fish feed are significantly different (*P* < 0.05): TN, TDN or NH_4_^+^-N concentrations with HT are the most, next with HP and the smallest with ZT; and actually these nutrients concentrations in the whole experimental period from HT fish feed are 1.17~1.52 times and 1.23~1.37 times the concentrations of HP and ZT, respectively for the same fish feed dosage. Average TN concentrations are 9.85, 7.20 and 5.36 mg L^−1^, average TDN concentrations are 7.93, 5.76 and 4.73 mg L^−1^, and average NH_4_^+^-N equilibrium concentrations are 6.63, 4.15 and 2.98 mg L^−1^ for HT 0.1 g, HP 0.1 g and ZT 0.1 g respectively, and corresponding concentrations with 0.2 g fish feed are almost twice their respective concentrations of treatments with 0.1 g fish feed. In reality, as shown in Table 1, ZT fish feed also contains the lowest crude protein, which may be due to the reason that ZT fish feed releases the smallest amount of nitrogen. In addition, similar to variations of TPP with time, TPN concentrations in Fig. 2(b,d) also fluctuate in low concentrations in all treatments during the whole period. Meanwhile, TPN concentrations are significantly different among the three different fish feed (*P* < 0.05).

As shown in Figs. 1 and 2, although the nutrients concentrations are significantly different in most experimental runs among HT 0.1 g, HP 0.1 g, ZT 0.1 g, HT 0.2 g, HP 0.2 g and ZT 0.2 g (*P* < 0.05), the nutrients’ proportions, namely, TDP:TP, PO_4_^3−^-P:TP, TPP:TP, TDN:TN, NH_4_^+^-N:TN and TPN:TN, are quite close after all nutrients concentrations reach their equilibrium concentrations, as shown in Table 2, for example, TDP is 84.48~91.95%, 88.80~94.90% and 80.91~90.93% of TP for HT, HP and ZT respectively. From the results in Table 2, the ratio of PO_4_^3−^-P to TP and NH_4_^+^-N to TN are obviously lower than those of TDP to TP and TDN to TN respectively because PO_4_^3−^-P and NH_4_^+^-N are only one part of them, respectively. Proportions of PO_4_^3−^-P and NH_4_^+^-N are in good agreement with Wu *et al*.’s results, and PO_4_^3−^-P and NH_4_^+^-N have high proportions of TP and TN ^43^, respectively. Butz and Ven-Cappell^59^ and Kibria *et al*.^35^ also believe that fish feed contained major phosphorus fraction in a labile form, namely, the total phosphorus in fish feed, the more the water-soluble phosphorus. Thus, according to released P (TP, TDP and PO_4_^3−^-P) and N (TN, TDN and NH_4_^+^-N) concentrations, we believed that HT contains the most nutrients, HP is next while ZT is the lowest in a comprehensive view. It is consistent with crude protein indicators of fish feed in general, ZT fish feed has the lowest crude protein level at 20%. Thus, based on trade-offs among feed price, feed efficiency, feed cost, feed quality, environmental impacts and so forth in aquaculture operations, we could improve protein bioavailability and design reasonable ratio of protein to energy to save protein and reduce nutrients emission.

**Table 2:** Nutrients proportions after nutrients reach their equilibrium concentrations.

Group	TDP:TP	PO_4_^3−^-P:TP	TPP:TP	TDN:TN	NH_4_^+^-N:TN	TPN:TN
HT	84.48~91.95%	81.98~88.42%	8.05~15.52%	79.24~85.96%	68.31~73.24%	14.04~20.76%
HP	88.80~94.90%	82.91~93.87%	5.10~11.20%	83.24~90.29%	57.08~62.18%	9.71~16.76%
ZT	80.91~90.93%	75.16~90.91%	9.07~19.09%	81.62~90.32%	59.32~78.90%	9.68~18.68%
MHT	24.64~36.55%	23.83~34.66%	63.45~75.36%	8.88~12.64%	0.53~4.68%	87.36~91.12%
MHP	28.92~37.71%	27.34~33.22%	62.29~71.08%	9.12~17.48%	0~5.18%	82.52~90.88%
MZT	30.23~43.39%	30.16~40.95%	56.61~69.77%	6.22~17.80%	0~7.46%	82.20~93.78%

HT, 0.1 g or 0.2 g fish feed of HT; HP, 0.1 g or 0.2 g fish feed of HP; ZT, 0.1 g or 0.2 g fish feed of ZT; MHT, *M. aeruginosa* + 0.1 g or 0.2 g fish feed of HT; MHP, *M. aeruginosa* + 0.1 g or 0.2 g fish feed of HP; MZT, *M. aeruginosa* + 0.1 g or 0.2 g fish feed of ZT.

### Effects of different fish feed on *M. aeruginosa* growth

#### Effects of different fish feed on *M. aeruginosa* densities

**Figure 3:**
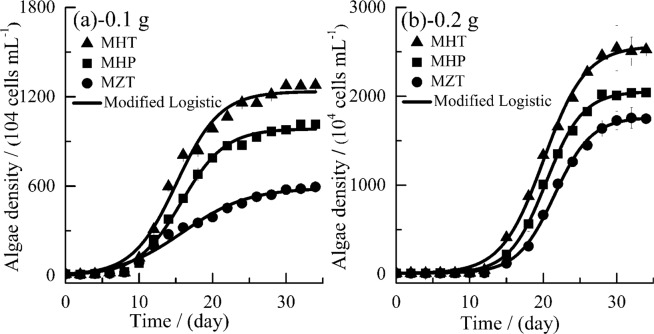
The growth of *M. aeruginosa* (MHT, *M. aeruginosa* + fish feed of HT; MHP, *M. aeruginosa* + fish feed of HP; MZT, *M. aeruginosa* + fish feed of ZT). Data shown is the mean ± SD of two independent measurements.

Fish feed contributes to abundant nutrient loads as discussed in the above, and it can effectively promote the growth of phytoplankton^28,43,60^. From Fig. 3, in the first few days of the experiment, algal cell densities increase very slowly due to their acclimation in fish feed medium with abundant nutrients in the medium. As time goes, *M. aeruginosa* cell densities increase very fast in the exponential phase (12~25 days) followed by a stable phase.

Not only fish feed dosage but also their quality affects algae growth greatly, and the algae densities’ rankings in Fig. 3 are in agreement with those rankings of nutrients concentrations generally. The order of algae densities from the three different fish feed is MHT 0.2 g (MHT 0.1 g) > MHP 0.2 g (MHP 0.1 g) > MZT 0.2 g (MZT 0.1 g) during the whole experimental period (Fig. 3), and the corresponding measured maximum algae density is 2526.1 (1278.9), 2042.0 (1016.4) and 1757.2 (595.2) 1 × 10^4^ cells mL^−1^, respectively. Two kinds of significant difference analysis of algae densities are conducted, namely, including and excluding lag phase, which indicates that the algae densities of MZT are significant different from those of MHT and MHP when excluding lag phase (*P* < 0.05), while they are not significantly different when including lag phase (*P* > 0.05), and this may be because the algae density is low and close to each other during the lag phase among the three different fish feed. In addition, fish feed dosage also has a significant effect on algae densities (*P* < 0.05).

Eutrophication is a major environmental problem induced by aquaculture activities, and algae densities reflect the level of eutrophication. Generally speaking, the lower the algae densities simulated by fish feed, the better the water quality is. Algae densities are coherent with released nutrients concentrations from fish feed and also consistent with nutritional indicators of fish feed in general. Thus, the above results imply that in order to protect aquaculture water environment, “environmentally friendly feed” are needed to both stimulate fish growth greatly and to lessen their effects on the water environment effectually in a balanced way. Meanwhile, new method is greatly needed to decrease the uneaten fish feed when throwing feed to fish manually and the uneaten fish feed also should be removed quickly before it releases nutrients to water.

In our study, both Fig. 3 and Table 3 show that the modified Logistic function can describe *M. aeruginosa* growth with good accuracy (*R*^2^ = 0.984~0.999) in agreement with the reported results^49^. Consistent with measured algae densities, ${N}_{max}$ and ${N}_{ave}$ (time-averaged algae density) of different fish feed are also in the order of MHT > MHP > MZT with the same fish feed dosage, and ${N}_{max}$ and ${N}_{ave}$ also increase with increasing dosages of fish feed. Specifically, the fitted *N*_*max*_ are 2557.32, 2044.95, 1753.91, 1232.98, 979.49 and 593.59 1 × 10^4^ cells (mL·d)^−1^ for MHT 0.2 g, MHP 0.2 g, MZT 0.2 g, MHT 0.1 g, MHP 0.1 g and MZT 0.1 g respectively, as shown in Table 3.

**Table 3:** Parameters of modified Logistic function describing algae growth. *a*, a constant; *r* (d^−1^), the intrinsic growth rate; ${N}_{max}$ (1 × 10^4^ cells mL^−1^), the maximum algae density; ${N}_{ave}$ (1 × 10^4^ cells mL^−1^), the average algae density; ${R}^{2}$, square of correlation coefficient; ${\mu }_{cmax}^{^{\prime} }$ (1 × 10^4^ cells (mL·d)^−1^), the maximal growth rate; ${\mu }_{cave}^{^{\prime} }$ (1 × 10^4^ cells (mL·d)^−1^), the average growth rate; ${\mu ^{\prime} }_{cmax}$ (1 × 10^4^ cells (mL·d)^−1^), the maximal specific growth rate; ${\mu }_{cave}$ (1 × 10^4^ cells (mL·d)^−1^), the average specific growth rate. Data were calculated according to corresponding equations.

Parameter	MHT 0.1 g	MHP 0.1 g	MZT 0.1 g	MHT 0.2 g	MHP 0.2 g	MZT 0.2 g
$a$	5.06	5.31	3.62	7.00	8.16	8.52
$r$	0.34	0.34	0.23	0.35	0.40	0.40
${N}_{max}$	1232.98	979.49	593.59	2557.32	2044.95	1753.91
${N}_{ave}$	687.70	529.74	308.78	1057.68	829.12	654.30
${R}^{2}$	0.988	0.996	0.984	0.998	0.999	0.998
${\mu }_{cmax}^{^{\prime} }$	103.57	82.52	33.69	223.13	204.50	173.64
${\mu }_{cave}^{^{\prime} }$	35.58	28.57	16.67	72.24	58.62	51.44
${\mu }_{cmax}$	0.31	0.29	0.31	0.31	0.34	0.34
${\mu }_{cave}$	0.14	0.14	0.12	0.15	0.14	0.14

#### Effects of different fish feed on the growth rate of *M. aeruginosa*

**Figure 4:**
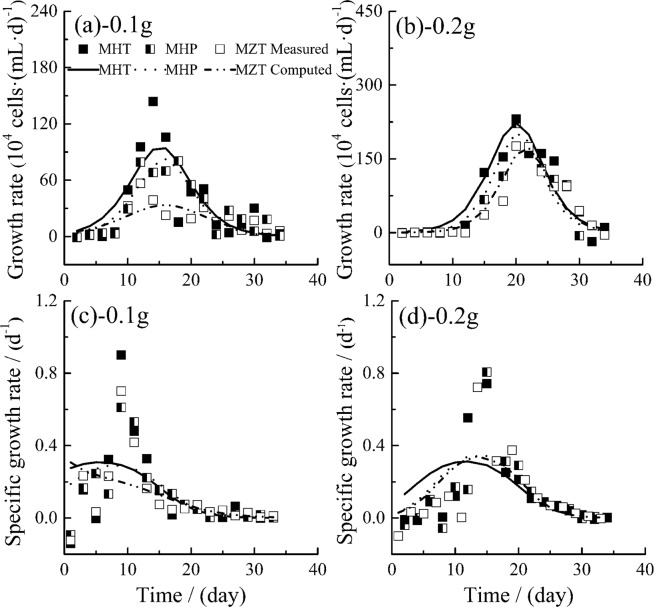
Variations of growth rates and specific growth rates of *M. aeruginosa* in fish feed with time (MHT, *M. aeruginosa* + fish feed of HT; MHP, *M. aeruginosa* + fish feed of HP; MZT, *M. aeruginosa* + fish feed of ZT). Data shown are average value of two independent measurements.

As shown in Fig. 4(a,b), both measured and computed growth rates in different groups all increase monotonously with time before they reached their maximal values, and then all decrease monotonously, which is consistent with Huang *et al*.’s study^49^. From Fig. 4(a,b) and correlation analysis, the computed growth rates agree reasonably well with measured ones with correlation coefficients (*R*) of 0.911, 0.954, 0.825, 0.970, 0.970 and 0.975 for MHT 0.1 g, MHP 0.1 g, MZT 0.1 g, MHT 0.2 g, MHP 0.2 g and MZT 0.2 g respectively, and all correlations are significant (*P* < 0.001). Although the analysis of significant difference shows that the fish feed quality does not have significant effects on growth rate (*P* > 0.05), maximal calculated growth rates (${\mu }_{cmax}^{^{\prime} }$) and averaged calculated specific growth rates of MHT are obviously the most, next those of MHP while those of MZT the smallest, as shown in Table 3.

#### Effects of different fish feed on the specific growth rate of *M. aeruginosa*

Correlation analysis between measured and computed specific growth rates is conducted, the correlation coefficients (*R*) between measured and computed specific growth rates in all groups range from 0.713 to 0.841 (*P* = 0.002~0.037) except in group MZT 0.1 g with *R* = 0.579 and *P* = 0.188. This indicates that Eq. (3) is reasonably well in describing specific growth rates of algae generally. In Fig. 4(c,d), the computed specific growth rates increase firstly, then decrease in general. In addition, both measured and computed specific growth rates among different qualities’ fish feed are quite close with the same fish feed dosage, significant difference analysis also shows that fish feed quality does not influence the specific growth rates significantly(*P* > 0.05). This is because the specific growth rate is defined as the growth rate relative to (divided by) the algae density (as described in Eq. (3)).

### Interaction of different fish feed and *M. aeruginosa* growth on nutrients concentrations

As discussed in 2.1, different quality of fish feeds has markedly different influence on released nutrients concentrations in general, that further affect algae growth. Wu *et al*. believe that in the presence of both algae and fish feed, nutrients releases were mainly controlled by fish feed dosage and algae utilization^43^. In the present study, not only fish feed dosage and algae utilization but also fish feed quality is taken into account to study the interaction of different fish feed and *M. aeruginosa* growth on nutrients concentrations.

#### Interaction of different fish feed and *M. aeruginosa* growth on phosphorus concentrations

**Figure 5:**
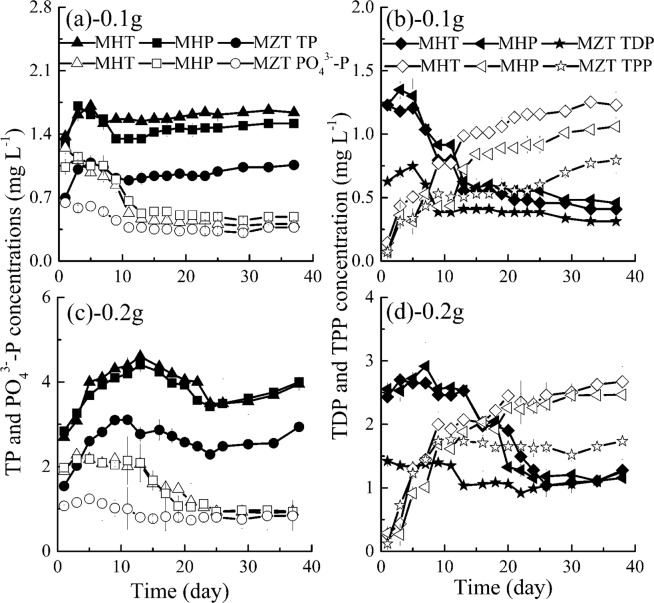
Variations of TP, TDP, TPP and PO_4_^3−^-P concentrations with time (MHT, *M. aeruginosa* + fish feed of HT; MHP, *M. aeruginosa* + fish feed of HP; MZT, *M. aeruginosa* + 0.1 g fish feed of ZT). Data shown is the mean ± SD of two independent measurements.

Figure 5 shows variations of TP, TDP, TPP and PO_4_^3−^-P concentrations with time in treatments with algae. From Fig. 5(a,b), some fluctuations of TP concentrations in treatments with algae were observed during the whole experimental period, and TP concentrations is not related to algae growth (*R* = −0.213~0.461, *P* = 0.072~0.928). Variations of PO_4_^3−^-P concentrations with time are similar to those of TDP, and both concentrations decrease gradually to minimal values, which have negative relationships with *M. aeruginosa* growth (*R* = −0.965~−0.623, *P* < 0.010 for PO_4_^3−^-P; *R* = −0.975~−0.539, *P* < 0.031 for TDP).The above variations of PO_4_^3−^-P and TDP with time in the present study are consistent with Zhou *et al*.’s^16^ and Wu *et al*.’s^43^ studies.

The bioavailability of phosphorus depends on the phosphorus speciation, and algae take up phosphorus predominantly in the form of free orthophosphate^35,61^. Zhou *et al*.’s results also show that the dissolved reactive phosphorus (mainly PO_4_^3−^-P) could be assimilated by algae at a higher velocity than other phosphorus forms^17^. As shown in Fig. 5, with the same fish feed dosage, any forms of P (TP, TDP and PO_4_^3−^-P) concentrations in MHT and MHP are close to each other, which are higher than those of MZT. There are significant differences only between MHT and MZT as well as between MHP and MZT for TP concentrations and also there is a significant difference between MHP and MZT for TDP concentrations (*P* < 0.05). However, if we compare maximal and averaged TP, TDP, PO_4_^3−^-P concentrations in the three different fish feed, they are actually quite different, and the most appears in MHT, and MHP is next while MZT is the smallest in general.

As shown in Fig. 5(b,d), TPP concentrations increase rapidly in the first 13 days then increase slowly in the following days. This is mainly related to initially released large quantities of phosphorus nutrients and uptake of PO_4_^3−^-P nutrients by algae. In Huang *et al*.’s^28^ study, TPP concentrations are closely related to the algae biomass, namely, variations of TPP concentrations with time are similar to those of algae biomass. Correlation analysis in the present study also shows that there are positive correlations between TPP concentrations and algae densities in most groups (*R* = 0.710~−0.917, *P* < 0.002) expect group MZT 0.2 (*R* = 0.349, *P* = 0.192). This is because TPP concentrations do not increase and even decrease since day 11 in group MZT 0.2. Meanwhile, consistent with algae density, the order of TPP concentrations is also MHT 0.2 g (MHT 0.1 g) > MHP 0.2 g (MHP 0.1 g) > MZT 0.2 g (MZT 0.1 g), and the corresponding average TPP concentrations is 1.94 (0.89), 1.78 (0.70) and 1.47 (0.52) mg L^−1^. However, quality or dosage has no significant effect on TPP concentrations in general (*P* > 0.05), which maybe because the difference of algae density among different quality of fish feeds are not significant especially during lag phase.

In addition, it is needed to point out that TP includes both extracellular P and intracellular P in treatments with algae, thus variations of TP concentrations with time in treatments with and without algae should be similar. However, we noted that, influenced by algae utilization and algae deposition, TP concentrations in groups with algae fluctuate and are lower than those in group without algae^43,62^.

#### Interaction of different fish feed and *M. aeruginosa* on nitrogen concentrations

**Figure 6:**
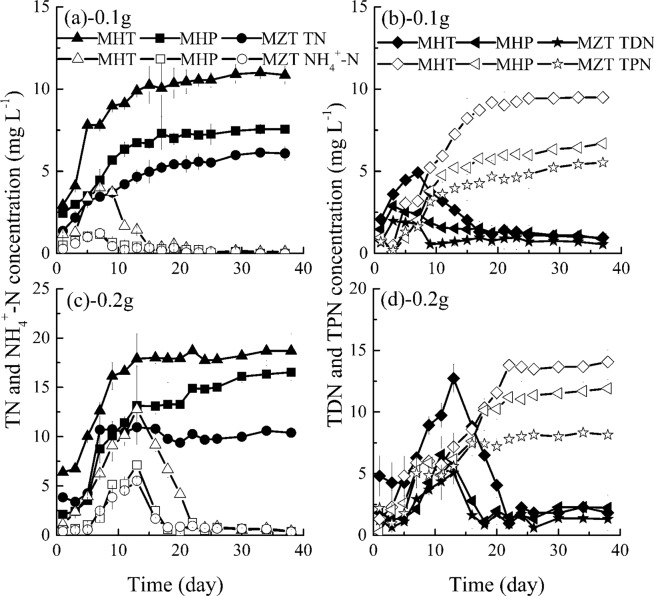
Variations of TN, TDN, TPN and NH_4_^+^-N concentrations with time (MHT, *M. aeruginosa* + fish feed of HT; MHP, *M. aeruginosa* + fish feed of HP; MZT, *M. aeruginosa* + fish feed of ZT). Data shown is the mean ± SD of two independent measurements.

From Fig. 6(a,c), TN concentrations in treatments with algae increase gradually in the first 15 days and then keep stable in the following days, the variations are consistent with those in treatments without algae. Meanwhile, algae densities are also related to TN concentrations released from fish feed in general (*R* = 0.616~0.908, *P* < 0.011), while the correlation coefficients are low in group MZT 0.2 with *R* = 0.357 (*P* = 0.175). Fish feed quality has significant influence on TN concentrations (*P* < 0.05), and the order of TN concentrations in groups is MHT > MHP > MZT in Fig. 6. Maximal TN concentrations are 11.00, 7.56 and 6.09 mg L^−1^, the average values are 9.10, 6.09 and 4.57 mg L^−1^ for MHT 0.1 g, MHP 0.1 g and MZT 0.1 g respectively. Meanwhile the corresponding TN concentrations almost double in treatments with 0.2 g fish feed in general.

NH_4_^+^-N is the main form of TDN also being the preferred form of nitrogen for algae growth^63^. From Fig. 6, both TDN and NH_4_^+^-N concentrations in treatments with algae increase to their maximal values firstly which is mainly affected by the release of TDN and NH_4_^+^-N from fish feed, then decrease in the following days affected by algal nutrients utilization generally. In general, correlation analysis indicates that there are negative relationships between algae densities and TDN concentrations (*R* = −0.887~−0.369, *P* = 0.001~0.159) and between algae densities and NH_4_^+^-N concentrations (*R* = −0.867~−0.504, *P* < 0.046). Different from the results in treatments without algae fish feed quality observes no significant effect on TDN and NH_4_^+^-N concentrations among MHT, MHP and MZT (*P* > 0.05), except that there is significant difference of TDN concentrations between MHT and MZT. Whereas, maximal and average values also show that MHT contains most TDN and NH_4_^+^-N concentrations, MHP next while MZT contains the lowest. Actually, NH_4_^+^-N concentrations have dropped to almost 0 mg L^−1^ in treatments with 0.1 g fish feed in the later period of algae growth and to 0.33~0.38 mg L^−1^ in treatments with 0.2 g fish feed (Fig. 6(a,c)).

In Fig. 6(b,d), TPN concentrations increase gradually in the first 20 days and then reach stable concentrations with time going in MHT, MHP and MZT. Consistent with TPP, TPN concentrations also have positive correlation with algae densities during the whole experimental period (*R* = 0.744~0.920, *P* < 0.001). Also, the order of TPN concentrations at the same time among different treatments is MHT 0.2 g (MHT 0.1 g) > MHP 0.2 g (MHP 0.1 g) > MZT 0.2 g (MZT 0.1 g), and the corresponding average TPN concentrations are 10.95 (6.77), 9.30 (4.50) and 7.19 (3.61) mg L^−1^. However, fish feed quality has no significant influence on TPN concentrations among all treatments with algae (*P* > 0.05), and this may be also because fish feed has no significant influence on algae densities when including the data in the lag phase (*P* > 0.05, *n* = 16).

Due to the effect of algae growth, the fractional composition in treatments with algae, as shown in Figs. 5 and 6 and Table 2, is different from that without algae, as shown in Figs. 1, 2 and Table 2. For example, due to the algae utilization, the ratio of TDN:TN is 8.88~12.64%, 9.12~17.48% and 6.22~17.80% for MHT, MHP and MZT respectively (in Table 2), which are largely lower than those of HT, HP and ZT mainly because of selective uptake of nutrients by algae.

#### Effectsof different fish feed on nutrients utilization by *M. aeruginosa*

Nutrients releases from HT, HP and ZT fish feed are different as discussed in 2.1, which further affect algae growth and nutrients utilization. In order to study the interaction between different fish feed and *M. aeruginosa* growth, nutrients utilization by algae is also explored. In Huang *et al*.’s^49^ and Goudar *et al*.’s^50^ studies, Logistic function is also used to simulate nutrients consumption versus incubation time and as follows:

4 $$\Delta C=\frac{\Delta {C}_{max}}{1+{e}^{{a}_{\bigtriangleup C}-{r}_{\Delta C}t}}$$

in which *t* is the incubation time (d), *ΔC* (i.e. △TDP, ΔPO_4_^3−^-P, ΔTDN and ΔNH_4_^+^-N) is consumed nutrient concentrations (difference of nutrients concentrations between without and with algae) at time *t* (mg L^−1^), $\Delta {C}_{max}$ is the maximum consumed nutrient concentrations, ${r}_{\bigtriangleup C}$ is the consumed rate constant (d^−1^) and ${a}_{\Delta C}$ is a constant.

**Figure 7:**
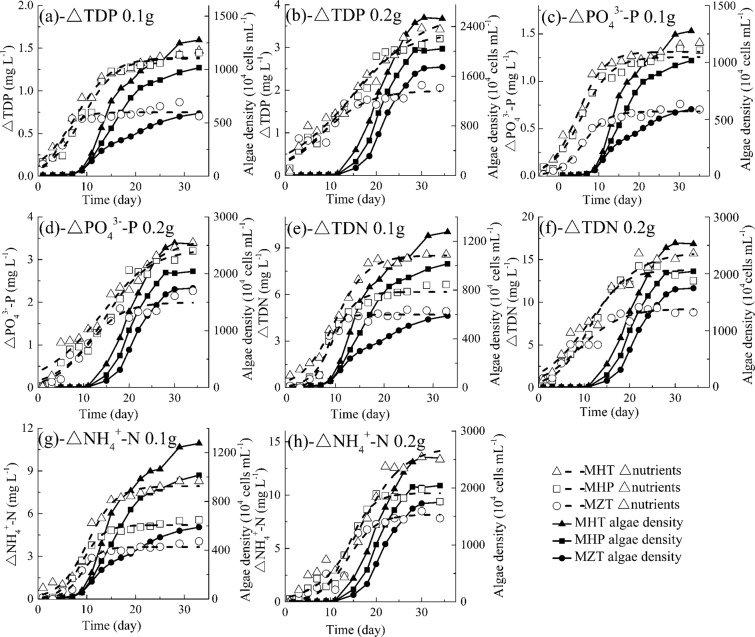
Variations of consumed TDP, PO_4_^3−^-P, TDN and NH_4_^+^-N concentrations with time (MHT, *M. aeruginosa* + fish feed of HT; MHP, *M. aeruginosa* + fish feed of HP; MZT, *M. aeruginosa* + fish feed of ZT). Data shown is the mean ± SD of two independent measurements.

As shown in Fig. 7, △TDP, △PO_4_^3−^-P, △TDN and △NH_4_^+^-N concentrations increase rapidly until it reaches their respective maximal consumed concentrations, then they remain stable. From Fig. 7 and Table 4, Eq.(4) can well describe variations of △TDP, △PO_4_^3−^-P, △TDN and △NH_4_^+^-N concentrations with time (${R}^{2}$ = 0.89~0.99), which is consistent with Kong *et al*.’s^55^ and Huang *et al*.’s^49^ study. In Table 4, it can also be founded that maximal calculated consumed TDP, PO_4_^3−^-P, TDN and NH_4_^+^-N concentrations ($\Delta {C}_{max}$) and averaged measured consumed concentrations ($\Delta {C}_{ave}$) in different treatments are in the order of MHT 0.2 g > MHP 0.2 g > MZT 0.2 g > MHT 0.1 g > MHP 0.1 g > MZT 0.1 g, for example, the corresponding *△C*_*max*_ of TDP is 3.85, 3.33, 1.99, 1.39, 1.38 and 0.75 mg L^−1^, respectively, this conforms to measured results. $\Delta {C}_{max}$ increases with increasing maximum density of *M. aeruginosa* (${N}_{max}$), which indicates that more algae need more nutrients to grow (Fig. 7). Correlation analysis also shows that there is a positive correlation between algae density and consumed TDP, PO_4_^3−^-P, TDN as well as NH_4_^+^-N concentrations with ${R}^{2}$ = 0.738~0.949, ${R}^{2}$ = 0.840~0.955, ${R}^{2}$ = 0.816~0.949, ${R}^{2}$ = 0.879~0.977, respectively. Meanwhile, fish feed quality has statistically significant effect on nutrient utilization if excluding the lag phase in general (*P* < 0.05) but no significant effect if including the lag phase (*P* > 0.05), and this is also because the algae density is close during the lag phase with different fish feed. In sum, the result implies that the nutrient utilization is dependent not only on the fish feed dosage but also on their quality.

**Table 4:** Parameters in Logistic function of consumed nutrients concentrations.

ΔTDP						
Parameter	MHT 0.1 g	MHP 0.1 g	MZT 0.1 g	MHT 0.2 g	MHP 0.2 g	MZT 0.2 g
*a*_Δ*C*_	2.03	2.62	2.32	1.94	2.27	1.59
*r*_Δ*C*_	0.26	0.29	0.51	0.13	0.17	0.19
$\Delta {C}_{max}$	1.39	1.38	0.75	3.85	3.33	1.99
${R}^{2}$	0.98	0.98	0.92	0.96	0.97	0.89
$\bigtriangleup {C}_{ave}$	1.01	0.95	0.64	1.96	1.88	1.40
**ΔPO**_**4**_^3−^-P						
*a*_Δ*C*_	2.92	2.89	3.70	2.15	2.94	2.83
*r*_Δ*C*_	0.37	0.33	0.41	0.15	0.20	0.26
$\Delta {C}_{max}$	1.31	1.26	0.68	3.55	3.23	2.00
${R}^{2}$	0.98	0.98	0.98	0.96	0.97	0.95
$\Delta {C}_{ave}$	0.95	0.88	0.47	1.89	1.71	1.27
**ΔTDN**						
*a*_Δ*C*_	3.06	4.29	6.11	2.14	3.07	1.97
*r*_Δ*C*_	0.30	0.44	0.73	0.19	0.28	0.22
$\Delta {C}_{max}$	8.52	6.16	4.70	15.87	13.79	9.25
${R}^{2}$	0.99	0.98	0.98	0.96	0.98	0.93
$\Delta {C}_{ave}$	5.61	4.11	3.38	9.74	8.82	6.36
**ΔNH**_**4**_^**+**^**-N**						
*a*_Δ*C*_	3.53	4.14	4.82	3.59	5.18	4.45
*r*_Δ*C*_	0.35	0.46	0.54	0.21	0.38	0.32
$\Delta {C}_{max}$	7.94	5.20	3.66	14.51	10.18	8.18
${R}^{2}$	0.98	0.99	0.98	0.95	0.97	0.95
$\Delta {C}_{ave}$	5.26	3.61	2.56	6.90	5.80	4.60

${a}_{\Delta C}$, a constant; ${r}_{\Delta C}$ (d^−1^), the consumed rate constant; $\Delta {C}_{max}$ (mg L^−1^), the maximum consumed nutrient concentrations; ${R}^{2}$, square of correlation coefficient; $\Delta {C}_{ave}$(mg L^−1^), the average consumed nutrient concentrations;. MHT 0.1 g, *M. aeruginosa* + 0.1 g fish feed of HT; MHP 0.1 g, *M. aeruginosa* + 0.1 g fish feed of HP; MZT 0.1 g, *M. aeruginosa* + 0.1 g fish feed of ZT; MHT 0.2 g, *M. aeruginosa* + 0.2 g fish feed of HT; MHP 0.2 g, *M. aeruginosa* + 0.2 g fish feed of HP; MZT 0.2 g, *M. aeruginosa* + 0.2 g fish feed of ZT. Data are obtained by fitting for two independent measurements.

In Tijani *et al*.’s study, both nitrogen and phosphorus utilization display a significant increase during the first 2~21 days, then enter a stationary phase on the 21st day and the utilization has an initial 48 h lag phase^64^. However, in the present study, as shown in Fig. 7, algae have consumed 0~1.5 mg L^−1^ of P and 0~7.5 mg L^−1^ of N in the lag phase of algae growth, and the nutrients utilization do not show clearly a lag phase even if the algae densities are low. This may be because the algae in Tijani *et al*.’s^64^ experiment do not experience the starvation just before their experiments.

## Conclusions

Three selected commercial compound fish feeds, HT, HP and ZT demonstrate different effects on released nutrients concentrations and *M. aeruginosa* growth because of their different qualities.

In treatments without *M. aeruginosa* (HT, HP, ZT), released P (TP, TDP, PO_4_^3−^P) and N(TN, TDN, NH_4_^+^-N) concentrations from different fish feeds are significantly different in general (*P* < 0.05), while there is no significant difference between HT and HP for released P concentrations (*P* > 0.05).

In treatments with *M. aeruginosa* (MHT, MHP and MZT), fish feed quality affects TP and TN concentrations significantly in general (*P* < 0.05). In addition, for most forms of consumed nutrients concentrations, the differences among all treatments excluding the lag phase are significant in most comparisons (*P* < 0.05), which suggests that the nutrient utilization is dependent on not only fish feed dosage but also fish feed quality. Maximum *M. aeruginosa* densities and growth rates in different fish feeds are also quite different, their orders are MHT > MHP > MZT with the same dosage.

In our study we experimentally studied the environmental effect of fish feed through incubator experiments without fish as a first try. Our preliminary results demonstrated that fish feed quality should be considered in terms of water environment protection.
